# Integrated preclinical and clinical development of mTOR inhibitors in pancreatic cancer

**DOI:** 10.1038/sj.bjc.6605819

**Published:** 2010-07-27

**Authors:** I Garrido-Laguna, A C Tan, M Uson, M Angenendt, W W Ma, M C Villaroel, M Zhao, N V Rajeshkumar, A Jimeno, R Donehower, C Iacobuzio-Donahue, M Barrett, M A Rudek, B Rubio-Viqueira, D Laheru, M Hidalgo

**Affiliations:** 1 The Sidney Kimmel Comprehensive Cancer Centre at Johns Hopkins and the Sol Goldman Pancreatic Cancer Research Center, The Johns Hopkins University, Baltimore, MD, USA; 2 Oncogenomics Laboratory, TGen Research Institute, Phoenix, AZ, USA; 3 Centro Integral Oncológico Clara Campal, Madrid, Spain; 4 Facultad de Medicina, Universidad-CEU San Pablo, Madrid, Spain; 5 Centro Nacional de Investigaciones Oncológicas, Melchor Fernandez Almagro 3, Madrid 28029, Spain

**Keywords:** pancreatic cancer, mTOR, p70S6K, temsirolimus, sirolimus

## Abstract

**Background:**

The purpose of this work was to determine the efficacy of inhibiting mammalian target of rapamycin (mTOR) in pancreatic cancer preclinical models and translate preclinical observations to the clinic.

**Methods:**

Temsirolimus (20 mg Kg^−1^ daily) was administered to freshly generated pancreatic cancer xenografts. Tumour growth inhibition was determined after 28 days. Xenografts were characterised at baseline by gene expression and comparative genomic hybridisation. Patients with advanced, gemcitabine-resistant pancreatic cancer were treated with sirolimus (5 mg daily). The primary end point was 6-month survival rate (6mSR). Correlative studies included immunohistochemistry assessment of pathway expression in baseline tumours, drug pharmacokinetics (PKs), response assessment by FDG-PET and pharmacodynamic effects in peripheral-blood mononuclear cells (PBMCs).

**Results:**

In all, 4 of 17 xenografts (23%) responded to treatment. Sensitive tumours were characterised by gene copy number variations and overexpression of genes leading to activation of the PI3K/Akt/mTOR pathway. Activation of p70S6K correlated with drug activity in the preclinical studies. Sirolimus was well tolerated in the clinic, showed predictable PKs, exerted pathway inhibition in post-treatment PBMCs and resulted in a 6mSR of 26%. No correlation, however, was found between activated p70S6K in tumour tissues and anti-tumour effects.

**Conclusion:**

Sirolimus activity in pancreatic cancer was marginal and not predicted by the selected biomarker.

Pancreatic cancer remains one of the most deadly cancers, ranking among the top causes of cancer-related deaths (Jemal *et al*, 2009). One of the reasons underlying the poor prognosis of this disease is the lack of effective systemic treatments. Patients with advanced pancreatic cancer are managed with gemcitabine-based combination chemotherapy with limited success (Moore *et al*, 2007). Attempts to develop new drugs in pancreatic cancer have, in general, focused on combination studies with gemcitabine, with very few single-agent screening trials being performed. Indeed, patients with gemcitabine-resistant disease have traditionally been considered too sick to participate in clinical trials and have been managed with palliative care. More recently, however, several phase II studies have shown that selected patients with gemcitabine-resistant pancreatic cancer can be safely treated with second-line chemotherapy (Gebbia *et al*, 2007; Kulke *et al*, 2007; Xiong *et al*, 2008; Wolpin *et al*, 2009). This observation opens the opportunity to test new agents in refractory patients in single-agent phase II studies. However, strategies to select potentially active drugs and candidate biomarkers for a more effective clinical development are needed.

The mammalian target of rapamycin (mTOR) pathway has emerged as an important candidate pathway for drug development (Rubio-Viqueira and Hidalgo, 2006; Meric-Bernstam and Gonzalez-Angulo, 2009). In recent years, several inhibitors of this pathway have been developed in cancer and some compounds have been approved for selected indications. The mTOR is downstream in the PI3K/Akt signalling pathway and is activated in response to growth factor receptor activation and nutrient stimulation. The mTOR regulates p70S6K and 4EBP1, having an important role in cell-cycle control and cell proliferation. Pancreatic cancer is characterised by several key genetic alterations such as activating mutations in the *KRAS* oncogene or inactivation in the *CDKN2A*/*INK4A* tumour suppressor gene that result in abnormal cell signalling and altered control of cell proliferation (Jones *et al*, 2008). Thus, pancreatic cancer is, in principle, an attractive tumour type to test mTOR inhibitors, and indeed, preclinical studies in established pancreatic cancer cell lines support this notion (Grewe *et al*, 1999; Agbunag and Bar-Sagi, 2004; Ito *et al*, 2006).

We have developed a set of freshly generated pancreatic cancer xenografts as a preclinical platform for preclinical screening and biomarker discovery in pancreatic cancer (Rubio-Viqueira *et al*, 2006). These tumours, which have been extensively characterised, represent the heterogeneity of the disease and retain the most important genetic features of the originator tumour (Rubio-Viqueira *et al*, 2006; Rubio-Viqueira and Hidalgo, 2009). In this study, we explored the activity of mTOR inhibition in pancreatic cancer with the goals to determine whether the observed activity warranted clinical development and to prioritise biomarkers that could be incorporated in clinical trials.

## Materials and methods

### Xenograft studies

Direct pancreatic cancer xenografts were generated as previously reported (Rubio-Viqueira *et al*, 2006). In this study we used 17 xenografts from the Hopkins PancXenoBank collection. Temsirolimus (Torisel, Wyeth Pharmaceuticals, Philadelphia, PA, USA) was administered intraperitoneally at 20 mg Kg^−1^ daily for 28 days as previously reported (Ito *et al*, 2006). Tumour size was evaluated two times per week by caliper measurements using the following formula: Tumour volume=(length × width^2^)/2. Relative tumour growth inhibition/regression was calculated as *T*/*C*=(*T*_i_−*T*_0_/*C*_i_−*C*_0_), *T*_i_ and *C*_i_ represent tumour size of treatment and control group at the end of experiments, respectively; *T*_0_ and *C*_0_ represent tumour size at initiation of experiments, respectively. *T*/*C*>0 represent growth inhibition, *T*/*C*<0 represents tumour regression. The research protocol was approved by the Johns Hopkins University Animal Care and Use Committee, and animals were maintained in accordance to guidelines of the American Association of Laboratory Animal Care.

### Microarray gene expression and array CGH profiling

Xenografts were profiled at baseline for gene expression using Affymetrix U133 Plus 2.0 gene arrays (Santa Clara, CA, USA) in duplicates as reported (Jimeno *et al*, 2008c). Similarly, array comparative genomic hybridization (CGH) was carried out as described (Barrett *et al*, 2004). Briefly, genomic DNA from normal and pancreatic cancer xenografts was fragmented and labelled according to published protocols (Wu *et al*, 2005). Labelled DNAs were hybridised to human Agilent 44A CGH microarrays consisting of ∼40 000 oligonucleotide probes (Agilent Technologies, Palo Alto, CA, USA) and scanned on an Agilent DNA microarray scanner. Raw log2 ratio data were calculated using Agilent Feature Extraction 9.1 software.

### Gene set enrichment analysis

Gene set analysis was performed using the Gene set enrichment analysis (GSEA) software V2.0.2 (http://www.broad.mit.edu/gsea) (Subramanian *et al*, 2005). Genes represented by more than one probe were collapsed using the Collapse Probes utility to the probe with the maximum value. Gene set permutations were performed 500 times for each analysis, and the pathway/gene set list is sorted by the Normalized Enrichment Score. We used the pathways defined by the Kyoto Encyclopedia of Genes and Genomes (KEGG) database to determine the rank-ordered pathway list for the xenografts. Human pathway annotations were downloaded from KEGG (August 2007 release), and 166 gene sets passed the gene set size filter criteria (min=10, max=500).

### ELISA

Levels of phosphorylated p70S6K were quantified at baseline using a solid-phase sandwich ELISA as per manufacture instructions (Immunoassay Kit, cat. no. KHO0581, Invitrogen, Camarillo, CA, USA).

### Clinical study

Patients with advanced pancreatic adenocarcinoma refractory to gemcitabine were eligible for this trial. Patients were required to have unidimensionally measurable disease and tumour tissue for immunohistochemistry (IHC) assessment or willingness to undergo a safe tumour biopsy. Other eligibility criteria included an Eastern Cooperative Oncology Group performance score 0–1, adequate haematological, renal and liver functions, including an absolute neutrophil count >1500 cells per mm^3^, haemoglobin >9 g per 100 ml, serum creatinine ⩽2 mg per 100 ml, bilirubin ⩽2 mg per 100 ml, ALT, AST, alkaline phosphatase ⩽5 times the upper limit of normal and triglycerides and total cholesterol <2 times the upper limit of normal.

Sirolimus was administered at a single oral flat dose of 5 mg per day continuously in an outpatient setting. A treatment cycle was of 28 days. Patients with grade 3 or 4 adverse events related to the study drug temporarily discontinued the treatment. They were asked to resume treatment on resolution of the toxic event to grade 0 or 1 at a reduced dose of 4 mg per day. Patients experiencing second grade 3 or 4 toxicity were to undergo a second dose reduction to 3 mg per day. In addition, patients who experienced symptomatic grade 2 toxicities or biochemical toxicity persisting for longer than 1 week had their daily dose of rapamycin decrease by one dose level to 4 mg per day. Patients requiring more than two dose reductions were taken off study. Patients had complete blood count and chemistry tests performed at baseline and every week for the first cycle and at every other week thereafter. Toxicity was graded according to the National Cancer Institute Common Toxicity Criteria for Adverse Events Version 3.0. Response to treatment was measured using FDG-PET-CT scan for every other cycle.

### Pharmacokinetic sampling, analytical assay and data analysis

Pharmacokinetic studies were performed after single and multiple doses during cycle 1 on days 1 and 28. Whole blood was collected in EDTA-containing tubes pre-treatment and at 1, 2, 4 and 6 h post-treatment on days 1 and 28. Trough samples were collected before drug administration on days 2, 3, 8, 15 and 22 of the first cycle and on day 1 of the second cycle. Samples were stored at −70°C or below. Sirolimus concentrations in whole blood were determined over a range of 0.5–200 ng ml^−1^ by a validated high-performance liquid chromatography with mass spectrometry detection (LC/MS/MS) method. Individual pharmacokinetic (PK) parameters were estimated by standard noncompartmental analysis using WINNonlin (Scientific Consultant, Apex, NC, USA) version 5.0 (Pharsight, Mountain View, CA, USA) (Gibaldi, 1982).

### Inmunohistochemistry of baseline tumour samples

Immunohistochemical labelling was performed using standard methods. Formalin-fixed 5 μm slides from paraffin-embedded tissue were deparafinised and stained with anti-phospho-p70S6K (Thr389) mouse monoclonal antibody (Cell Signaling Technologies). Immunolabelling was detected as per kit instructions (Ventana IVIEW Detection Kits, cat. no. 760091, Ventana). The intensity of the staining was determined using *H*-scores, a composite measure of intensity × proportion of staining cells.

### Correlative studies in peripheral-blood mononuclear cells

Levels of total and phosphorylated [pT389] p70S6K in peripheral-blood mononuclear cells (PBMCs) at baseline and at 6 h after the first sirolimus dose were quantified using a solid-phase sandwich ELISA as per manual instructions (Invitrogen Immunoassay kit, cat. no. KHO0581). The ratio of phospho/total p70S6K at 6 h after the first dose of sirolimus was normalised to baseline level and expressed as a percentage.

### Statistical considerations

The primary end point of the clinical trial was the proportion of patients surviving at 6 months after treatment commencement (6-month survival rate (6mSR)). The expected 6mSR with standard of care in the second-line setting is 30%, and on the basis of the preclinical study, a 25% positive outcome was expected (Rothenberg *et al*, 1996). The sample size was determined to detect an improvement in 6mSR from 30 to 50%. With 31 patients enroled and a one-side *α* of 0.05, the study has a 76% power to detect this difference. The secondary objectives included: (a) to evaluate the relationship between baseline phospho-p70S6K expression by IHC and clinical outcome; (b) to characterise the toxicity and PKs of sirolimus in this patient population and; (c) to determine the pharmacodynamic effects of the agent on p70S6K activation in PBMCs. To test whether activation of PI3K/Akt/mTOR pathway was correlated with survival in patients treated with sirolimus, a Fisher's exact test was performed to determine the relation between phospho-p70S6K levels at baseline and 6mSR. Differences between PK parameters during sampling periods were compared by a Wilcoxon matched-pairs signed-rank test. All PK parameters are reported as mean±standard deviation unless otherwise noted. Pearson's correlation coefficient or Mann–Whitney *U*-tests were used to assess correlations between exposure (*C*_max_ or AUC) and exploratory PD end points (that is, PBMC phospho-p70S6K, survival and PET-CT response). These tests were performed using JMP Statistical Discovery software (version 4.0.4; SAS Institute, Cary, NC, USA) or SPSS version 16 (SPSS, Chicago, IL, USA).

## Results

### Tumour growth inhibition with temsirolimus in direct pancreatic cancer xenografts correlated with pathway activation

**Figure 1:**
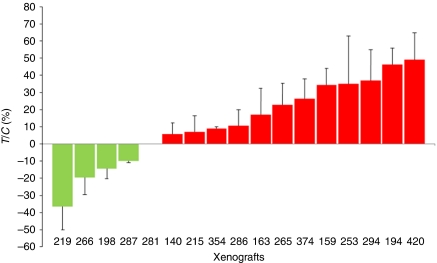
Tumour growth inhibition (*T*/*C*) in 17 direct pancreatic cancer xenografts treated with temsirolimus. Four xenografts showed tumour regression (negative *T*/*C*s). Bars represent standard deviation.

**Figure 2:**
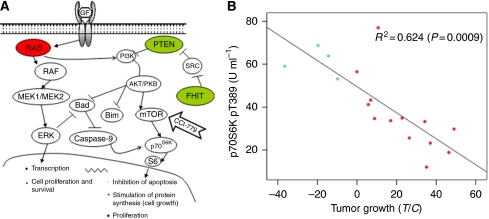
(**A**) Sensitive xenografts had focal gains in *RAS*, or homozygous deletions in *PTEN* or *FHIT*, potentially leading to pathway activation. Red colour represents gene copy number gains. Green colour represents gene copy number losses. (**B**) Tumour regression after treatment with temsirolimus in direct pancreatic cancer xenografts was correlated with baseline phospho-p70S6K. Green dots represent xenografts that experienced tumour regression after temsirolimus treatment. Red dots represent xenografts that experienced tumour growth.

We observed tumour regression in four xenografts treated with temsirolimus (Figure 1). Assessment of gene copy number variations in these tumours by CGH array showed gene copy number variations that could potentially lead to PI3K/Akt/mTOR pathway activation. Xenografts Panc219 and Panc198 had focal gains in *NRAS* and *KRAS*, respectively, whereas Panc266 and Panc287 had a homozygous deletion of *FHIT* or *PTEN*, respectively (Figure 2A). These genetic abnormalities were not, however, specific because some of the resistant tumours also had similar genetic alterations such a *FHIT* losses and *AKT* amplification. Supplementary Table 1 summarises the genomic alterations observed in these tumours.

To gain further insight into gene pathways that might predict response to temsirolimus, we performed GSEA on the gene expression profiles of both sensitive and resistant cases. From the GSEA results, there were 8 and 18 pathways with *P*<0.01 and false discovery rate <20% enriched in the sensitive and resistant cases, respectively (Table 1). Five cancer (chronic myeloid leukaemia, glioma, pancreatic, prostate and renal cell cancer) pathways were among the pathways enriched in the sensitive cases. We found 32 common core genes that were upregulated in more than two pathways enriched in the sensitive cases (Supplementary Table 2). Many of these common core genes (*AKT3*, *BRAF*, *EGFR*, *FGFR1*, *IGF1R*, *PIK3CA*, *PIK3R3*, *PDGFA* and *PDGFB*) were related to the PI3K/Akt/mTOR pathway.

To further explore whether those genetic changes result in pathway activation, we measured phospho-p70S6K in the 17 xenografts using an ELISA test as a downstream read out of pathway activation. As shown in Figure 2B, there was a significant correlation between tumour regression after treatment with temsirolimus and baseline activation of p70S6K.

From these studies, we concluded that mTOR inhibitors exerted distinct anti-tumour effects in pancreatic cancer xenografts that were characterised by heightened activation of the PI3K/Akt/mTOR pathway. To validate this hypothesis, we conducted a phase II trial with the mTOR inhibitor sirolimus in patients with advanced pancreatic cancer, integrating measurement of phospho-p70S6K activation as a predictor of response.

### General clinical results

A total of 31 patients, whose pertinent characteristics are listed in Table 2, were enroled in this trial. All patients had previously progressed to a gemcitabine-containing regimen, including 22 patients who had received previous chemotherapy for metastatic disease.

**Figure 3:**
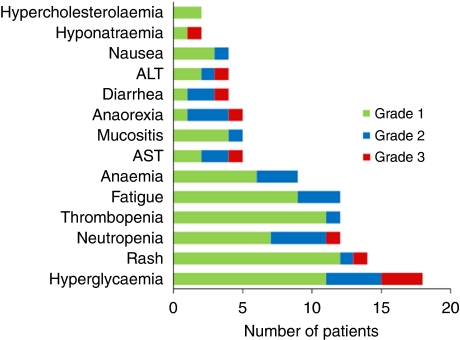
Treatment-related toxicities (*n*=31).

Overall treatment was well tolerated. The principal toxicities are summarised in Figure 3. Most of the adverse events were grade 1 (67%), 24% were grade 2, 9% were grade 3 and there was no grade 4 adverse events. The most common grade 3 adverse event was hyperglycaemia, observed in 10% of the patients. Although 22 (75%) patients had already received chemotherapy for metastatic disease before inclusion in this study, only one patient developed a grade 3 haematological adverse event (neutropenia). One patient developed a grade 3 increase in AST/ALT levels on cycle 1, day 15; treatment was stopped for 1 week with subsequent normalisation of liver function tests. One patient was admitted because of grade 3 enterocolitis on cycle 1, day 6; treatment was discontinued and the patient was discharged 4 days later. Other grade 3 toxicities were hyponatremia and rash in one patient (3%) each.

There were no objective responses in this trial. Four patients (13%) had stable disease (SD) at the 2-month follow-up evaluation. The 6mSR for the overall population was 26% (95% CI 13–45% Supplementary Figure 1).

### PI3K/Akt/mTOR/pathway activation and clinical outcome

We assessed baseline levels of phospho-p70S6K in 22 out of 31 patients. In contrast to the preclinical findings, there was no correlation between baseline levels of phospho-p70S6K by IHC and clinical outcome.

### PK analysis

PK data were evaluable in 30 patients. The PK profile of sirolimus was characterised by rapid absorption and a slow elimination phase after oral administration. By assessment of pre-treatment trough concentrations, steady state was reached by day 8. Accumulation was noted as there was an increase in sirolimus exposure (*C*_max_ 15.5±9.2 ng ml^−1^ (*n*=30) *vs* 24.8±10.4 ng ml^−1^ (*n*=20) from day 1 to day 28; and day 1 AUC_inf_ 234.5±121.5 ng h ml^−1^ (*n*=22) *vs* day 28 AUC_0–24 h_ 388.6±129.1 ng h ml^−1^ (*n*=15); (*P*<0.05)). There was a statistically significant increase in the day 28 half-life (10.63±2.57 h (*n*=22) *vs* 20.67±5.12 h (*n*=14) on days 1 and 28, respectively; *P*<0.05) and decrease in the apparent systemic clearance (28.6±17.3 l h^−1^ (*n*=22) *vs* 13.6±4.0 l h^−1^ (*n*=14) on days 1 and 28, respectively; *P*<0.05). Sirolimus showed extensive distribution in excess of blood volume (Vz/F 414.6±206.8 l (mean±s.d.; *n*=22) and 415.7±200.4 l (*n*=14) on days 1 and 28, respectively). The steady-state pre-treatment trough concentration was 12.6±5.1 ng ml^−1^ (*n*=28). No correlation was found between PK parameters clinical outcome and PD markers.

### PD evaluation

**Figure 4:**
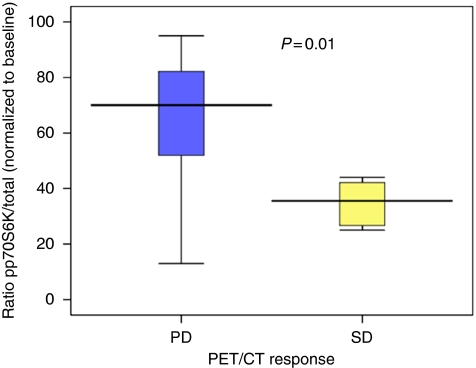
Activity of p70S6K at 6 h after first dose of sirolimus predicts stable disease on PET-CT evaluation. Patients with stable disease as shown by PET-CT on restaging at 8 weeks had greater inhibition of phospho-p70S6K in PBMC at 6 h than those with disease progression (*P*=0.01).

Patients with SD, as shown by PET-CT, had statistically significantly greater inhibition of p70S6K in PBMC at 6 h after treatment, as shown by a higher decrease in the ratio of phospho/total p70S6K (35 *vs* 63; *P*=0.01). Thus, activated phospho-p70S6K levels at 6 h were predictive for PET-CT response at 8 weeks (Figure 4).

## Discussion

This study aimed to determine the therapeutic role of inhibiting mTOR in preclinical models of pancreatic cancer and in patients with this disease. The mTOR inhibitor, temsirolimus, induced tumour regressions in 4 of 17 (23%) freshly generated pancreatic cancer xenografts that were, in conjunction, characterised by genetic alterations, leading to an increased activation in the PI3K/Akt/mTOR pathway. As a single agent, sirolimus resulted in a 26% 6mSR in patients with previously treated pancreatic cancer, but did not result in tumour regressions in any patients. Contrary to our hypothesis, there was no indication that patients with higher activation of the pathway, as measured by the selected biomarker in this trial, did better.

The role of preclinical models in cancer drug development continues to evolve. In the era of cytotoxic agents, models were used to show tumour growth inhibition in a randomly selected group of rapidly growing xenograft models (Berger *et al*, 1990). More recent efforts, however, include testing agents in larger collection of xenografts representing a disease of interest, not only to gauge potential activity but also to understand predictors of efficacy (Perez-Soler *et al*, 2000; Rubio-Viqueira *et al*, 2006; Houghton *et al*, 2007; Jimeno *et al*, 2008a). With this goal in mind, we and others have started conducing large-scale phase II-like preclinical studies in pancreatic cancer. In this work, we followed such an approach to test the activity of mTOR inhibitors in pancreatic cancer.

One important consideration in this work, however, is that the level of activity in preclinical models that predict clinical efficacy is not established. Classically, a *T*/*C* of 40% has been considered supportive of anti-tumour efficacy and used as a threshold to move drugs to the clinic. This criterion, in our opinion, is too unrestrictive and overestimates the expected clinical results. Indeed, if one applies the commonly used RECIST clinical criteria of response, a *T*/*C* of 40% would be disease progression. We have therefore applied a more restrictive criterion and consider activity if there is a tumour regression. The current situation is that there are thousands of anti-cancer agents available but yet very little work in the clinic. A more selective preclinical approach is needed to prioritise which drugs to develop in patients. Efforts to better establish levels of preclinical efficacy that predict positive clinical outcome are, indeed, needed.

Another important, and not established, question is which level of preclinical activity is required to justify conducting a clinical trial. It is remarkable to note the important parallelisms between the preclinical and clinical study with ∼25% cases meeting the pre-specified primary objective in both studies. Notwithstanding that our patients were very heavily pretreated, a 25% 6mSR in the second-line pancreatic cancer is low and does not warrant further development of this drug as a single agent in this disease unless a predictive biomarker is identified. This has been indeed the finding of another recently published trial (Wolpin *et al*, 2009). In retrospect, we should have established a higher threshold of activity in the preclinical study before advancing the agent to clinical development. Given the larger number of available agents in clinical development and the limited resources, we propose that only agents with preclinical activity significantly greater than the clinical activity of interest are selected for clinical development.

The second major goal of this work was to identify biomarkers of activity. The preclinical data show that tumour regression after treatment with temsirolimus was limited to xenografts with high activation of the PI3K/Akt/mTOR pathway, as shown by high activation of p70S6K. There is a whole body of literature supporting the role of *PTEN* losses in activation of the PI3K/AKT/mTOR pathway in a variety of tumours including prostate, breast and glioma (Li *et al*, 1997). Our finding that 1 of 17 (5%) xenografts had *PTEN* deletion is in consonance with previous evidence that *PTEN* losses are infrequent in pancreatic cancer (Okami *et al*, 1998). The fragile histidine triad (*FHIT*) gene, a tumour suppressor, is lost in most malignancies, including pancreatic cancer (Simon *et al*, 1998; Huebner and Croce, 2001). Loss of *FHIT*, leads to increase AKT activity both *in vitro* and *in vivo* (Semba *et al*, 2006). These findings are supported by the results from the GSEA, showing that sensitive xenografts were enriched in pathways with high content of genes involved in the PI3K/Akt/mTOR pathway. Thus, the striking correlation between the drug activity and pathway activation, as measured by phospho-p70S6K, a downstream mediator of the pathway, is expected on the basis of the current knowledge of this pathway.

Although the overall level of activity of mTOR inhibitors in pancreatic cancer was modest, the finding that the activity could be linked to a biomarker was critical to support the conduction of the clinical study. If the 25% of patients who are sensitive can be identified upfront, the clinical development of the drug in pancreas cancer is feasible and likely to be successful. Unfortunately, we did not observe such a relationship in the clinical trial. Several factors can be considered to explain this issue. First, it could be that the concentrations of sirolimus achieved in patients are inferior to those achieved in mice. However, we have used the maximum tolerated dose of sirolimus based on data from a previous phase I study published by our group (Jimeno *et al*, 2008b). Second, PDA is characterised by an intense desmoplastic reaction that may lead to decreased intratumoural concentrations of sirolimus and thus explain lack of activity (Olive *et al*, 2009). Third, it could be that the biomarker selected, that is, activation of p70S6K, is not valid. We doubt, however, this is the reason. The notion that activation of the PI3K/Akt/mTOR pathway leads to p70S6K activation is supported by multiple studies (Hennessy *et al*, 2005; Riemenschneider *et al*, 2006). In addition, several trials with mTOR inhibitors suggest that this is a candidate marker (Galanis *et al*, 2005; Cloughesy *et al*, 2008). More likely, the negative results are because of trial design and technical reasons. We have used an IHC technique applied to archival tissues and we really do not know the preservation of the phosphorylated antigen over time in these materials. This is a recurrent problem in clinical trials that relay on archival tissue for which no satisfactory solution has been proposed. In addition to this, other investigators have questioned the performance of the specific antibody we used (anti-Thr389 S6K) for reliable quantification of the activity of p70S6K on paraffin sections (Cloughesy *et al*, 2008). We assessed p70S6K in primary tumours and treated metastatic disease. We do not know whether the process of generating tumour metastasis results in variations in this biomarker. In retrospect, a better strategy would have been to perform a fresh biopsy from a metastatic site and to use the same ELISA method that we have used in the preclinical study. It is clear that this approach adds morbidity, complexity and costs, but it may at the end be the only strategy to answer these questions. Finally, it could just mean that the preclinical model is not predictive at all of clinical activity and what is learnt in one does not predict what happens in the other.

In summary we have integrated a preclinical and clinical trial of mTOR inhibitors in pancreatic cancer. The agent resulted in ∼25% of cases achieving a positive response. In the preclinical study, activity was linked to pathway activation. This finding, however, was not observed in the clinic. The most likely explanation for this negative result is technical in nature, but other factors cannot be ruled out. In the absence of a well-defined biomarker to select patients, the overall level of activity does not suggest that these drugs as single agents would be effective in this disease.

## Figures and Tables

**Table 1:** Gene pathways enriched in temsirolimus-sensitive and -resistant xenografts as per KEGG classification

**Name**	**KEGG pathway**	**Size**	**NES**	***P***	**FDR**
*Pathways enriched in the sensitive cases (*P*<0.01)*					
HSA03010	Ribosome	54	2.41	0.000	0.00
HSA05220	Chronic myeloid leukaemia	76	1.86	0.000	0.04
HSA04520	Adherens junction	79	1.83	0.000	0.03
HSA05214	Glioma	64	1.74	0.000	0.06
HSA05212	Pancreatic cancer	73	1.68	0.000	0.08
HSA05215	Prostate cancer	88	1.58	0.008	0.14
HSA04350	TGF-*β* signalling pathway	87	1.57	0.000	0.13
HSA05211	Renal cell carcinoma	69	1.52	0.007	0.15
					
*Pathways enriched in the resistant cases (*P*<0.01)*					
HSA00590	Arachidonic acid metabolism	52	−1.97	0.000	0.00
HSA00260	Glycine, serine and threonine metabolism	45	−1.97	0.000	0.00
HSA00591	Linoleic acid metabolism	35	−1.85	0.000	0.02
HSA00280	Valine, leucine and isoleucine degradation	43	−1.80	0.000	0.03
HSA03320	PPAR signalling pathway	66	−1.78	0.000	0.03
HSA00910	Nitrogen metabolism	23	−1.77	0.006	0.03
HSA00252	Alanine and aspartate metabolism	32	−1.76	0.000	0.03
HSA00450	Selenoamino acid metabolism	28	−1.75	0.000	0.03
HSA00051	Fructose and mannose metabolism	42	−1.66	0.009	0.07
HSA00710	Carbon fixation	22	−1.62	0.009	0.08
HSA00062	Fatty acid elongation in mitochondria	10	−1.62	0.010	0.08
HSA00970	Aminoacyl-tRNA biosynthesis	37	−1.59	0.006	0.10
HSA00620	Pyruvate metabolism	42	−1.58	0.009	0.10
HSA00562	Inositol phosphate metabolism	50	−1.58	0.003	0.09
HSA00650	Butanoate metabolism	44	−1.54	0.009	0.12
HSA00010	Glycolysis/gluconeogenesis	62	−1.49	0.009	0.16
HSA04020	Calcium signalling pathway	175	−1.47	0.008	0.17
HSA04080	Neuroactive ligand–receptor interaction	251	−1.46	0.003	0.17

Abbreviations: FDR=false discovery rate; KEGG=Kyoto Encyclopedia of Genes and Genomes; NES=normalized enrichment score; *P*=*P*-value; TGF=transforming growth factor.

**Table 2:** Patient's characteristics

**Characteristics**	**Number**	**Percentage**
*Age* (years)		
Median	64	
Range	39–77	
		
*Sex*		
Male	21	68
Female	10	32
		
*ECOG PS*		
0	12	39
1	19	61
		
*Type of surgery*		
Whipple	11	35
Pilorus preserving	4	13
		
No previous surgery	16	52
Previous perioperative chemoradiation	11	
Previous perioperative chemotherapy	4	
		
*Previous chemotherapy for metastatic disease*		
GEM-Tarceva	6	25
GEM	5	21
GEM-XELODA	3	12.5
GEM-CDDP	3	12.5
GEM-LOHP	3	12.5
GEM-CPT11	1	4
GEM-XELODA-Txt	1	4
GEM-Avastin	1	4
XELODA-Avastin-Tarceva	1	4
		
*Serum Ca 19-9 (U ml* ^*−1*^ *)*		
Median	11 048	
Range	4–82 808	

Abbreviations: CDDP=cisplatin; CPT-11=irinotecan; ECOG PS=Eastern Cooperative Oncology Group performance score; GEM=gemcitabine; LOHP=oxaliplatin; Txt=taxotere.
