# Supplementary figures and images for: Integrated preclinical and clinical development of mTOR inhibitors in pancreatic cancer
Source: Br J Cancer. 2010 Jul 27;103(5):649–55. doi: 10.1038/sj.bjc.6605819 (PMC2938261; doi:10.1038/sj.bjc.6605819)

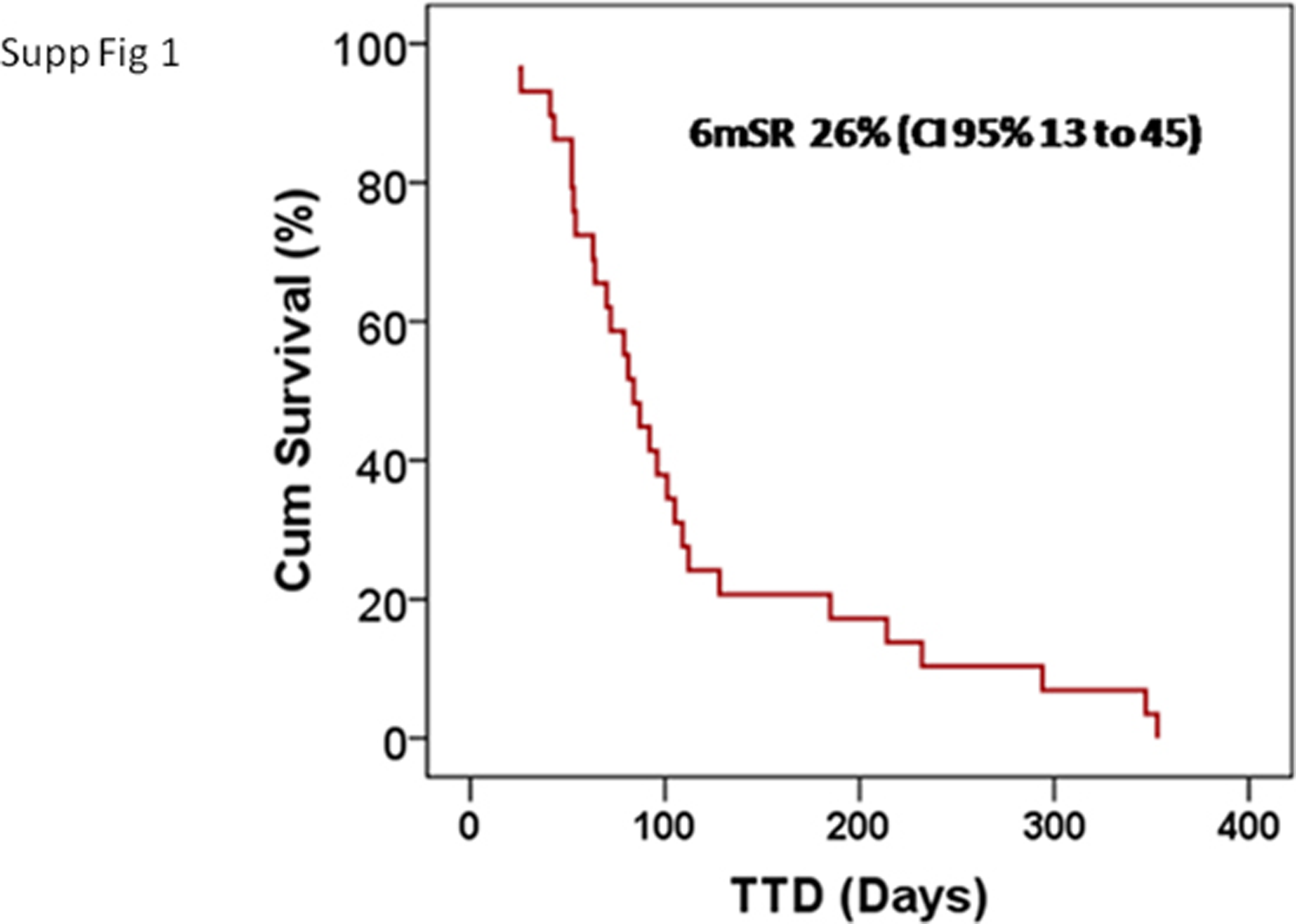

Supplement: Supplementary Figure S1 [file 6605819x1.tif]

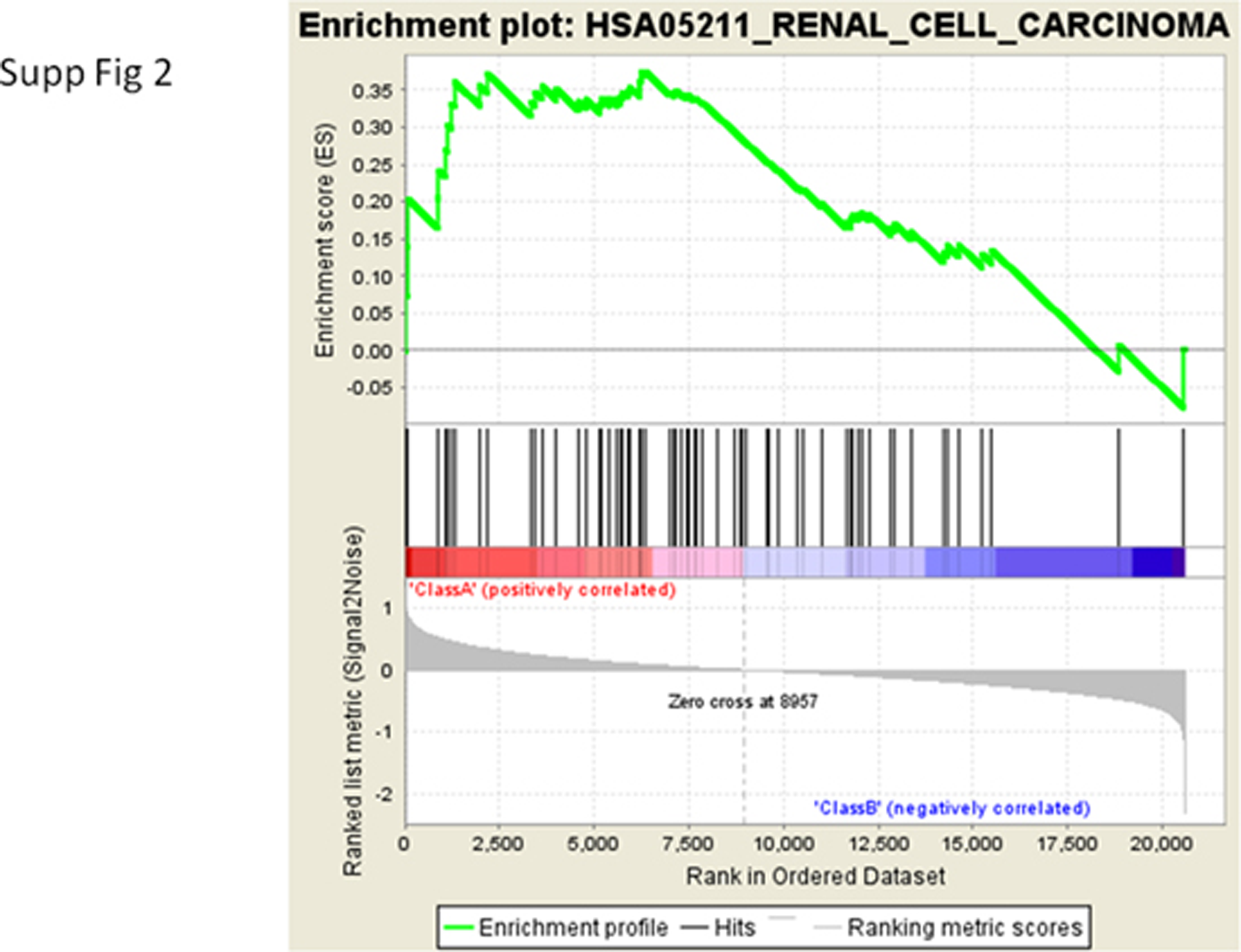

Supplement: Supplementary Figure S2 [file 6605819x2.tif]
