# Supplementary material for: Integrated preclinical and clinical development of mTOR inhibitors in pancreatic cancer
Source: Br J Cancer. 2010 Jul 27;103(5):649–55. doi: 10.1038/sj.bjc.6605819 (PMC2938261; doi:10.1038/sj.bjc.6605819)
Supplement: Supplementary Figures Legends [file 6605819x3.doc]

Supplementary Figure Legends

**Supplementary Figure 1**. Survival curve for patients treated with sirolimus

**Supplementary Figure 2**. Enrichment plot for renal cell carcinoma pathway

GSEA is an analytical tool that give us statistical measurements of what gene pathways are enriched when we compared different gene expression profiles (i.e. sensitive vs. resistant to temsirolimus). For this purpose we used the gene pathways as defined by KEGG (Kyoto Encyclopedia of genes and genomes). This is publicly available and it was our reference data base. GSEA compares the gene expression of the two groups that we have previously defined and generates a ranked order list of genes (red to blue bar in the middle section of the figure). As an example, at the top of the list (red color in the bar) we will find the genes that are upregulated in xenografts sensitive to temsirolimus (or downregulated in resistant xenografts), at the bottom of the list (blue color in the bar) we will find genes downregulated in xenografts sensitive to temsirolimus (or upregulated in resistant xenografts). The primary result of the gene set enrichment analysis is the enrichment score (ES) (top section of the figure). GSEA calculates the ES by walking down the ranked-ordered list of genes, increasing a running-sum statistic when a gene is in the gene set and decreasing it when it is not. If the genes contained in a pathway are overrepresented at the top or at bottom of the rank ordered list of genes, then the pathway is overrepresented in one of the two phenotypes. If the genes from a particular pathway are distributed homogeneously across the rank ordered list of genes then the pathway is not enriched in any of our two phenotypes
