# Supplementary material for: Integrated preclinical and clinical development of mTOR inhibitors in pancreatic cancer
Source: Br J Cancer. 2010 Jul 27;103(5):649–55. doi: 10.1038/sj.bjc.6605819 (PMC2938261; doi:10.1038/sj.bjc.6605819)
Supplement: Supplementary Table S1 and S2 [file 6605819x4.doc]

**Supplementary Tables**

**Supplementary Table 1: Baseline Gene Copy Number Variations in Xenografts Treated with Temsirolimus.**

| Xenograft | Focal Gains | Homozygous Losses |
| --- | --- | --- |
| 219 | N-Ras |  |
| 266 |  | FHIT |
| 198 | K-Ras |  |
| 287 |  | PTEN, SMAD4, FGR2, CDKN2A |
| 281 |  | CDKN2A |
| 140 | ERBB2, c-MYC |  |
| 215 | ERBB2, PIP5K2B, PIK4CA,EIF1 | FHIT |
| 354 | K-Ras, R-Ras, c-MYC, TYK2 | CDKN2A |
| 286 |  |  |
| 163 |  | CDKN2A |
| 265 | AKT2, K-Ras | CDKN2A |
| 374 | PIK4CA, AKT2, TESK1, TOP3B | FHIT |
| 159 |  | CDKN2A |
| 253 | AKT2, CDH2, TESK1 | PCDH9 |
| 294 | ERBB2, ETV4, TOB1 | TGFBR2, CDKN2A |
| 194 |  | SMAD4, CDKN2A |

**Supplementary Table 2: Common Core Genes Expressed in Xenografts Sensitive to Temsirolimus.**
